# A promising deep learning-assistive algorithm for histopathological screening of colorectal cancer

**DOI:** 10.1038/s41598-022-06264-x

**Published:** 2022-02-09

**Authors:** Cowan Ho, Zitong Zhao, Xiu Fen Chen, Jan Sauer, Sahil Ajit Saraf, Rajasa Jialdasani, Kaveh Taghipour, Aneesh Sathe, Li-Yan Khor, Kiat-Hon Lim, Wei-Qiang Leow

**Affiliations:** 1 Yong Loo Lin School of Medicine, National University Singapore, Singapore, Singapore; 2 Department of Anatomical Pathology, Singapore General Hospital, 20 College Road, Singapore, 169856 Singapore; 3 Qritive Pte. Ltd., Singapore, Singapore; 4 Duke-NUS Medical School, Singapore, Singapore; 5 School of Biological Sciences, Nanyang Technological University, Singapore, Singapore

**Keywords:** Computational models, Image processing, Machine learning, Pathology, Cancer imaging, Colon cancer, Colon cancer

## Abstract

Colorectal cancer is one of the most common cancers worldwide, accounting for an annual estimated 1.8 million incident cases. With the increasing number of colonoscopies being performed, colorectal biopsies make up a large proportion of any histopathology laboratory workload. We trained and validated a unique artificial intelligence (AI) deep learning model as an assistive tool to screen for colonic malignancies in colorectal specimens, in order to improve cancer detection and classification; enabling busy pathologists to focus on higher order decision-making tasks. The study cohort consists of Whole Slide Images (WSI) obtained from 294 colorectal specimens. Qritive’s unique composite algorithm comprises both a deep learning model based on a Faster Region Based Convolutional Neural Network (Faster-RCNN) architecture for instance segmentation with a ResNet-101 feature extraction backbone that provides glandular segmentation, and a classical machine learning classifier. The initial training used pathologists’ annotations on a cohort of 66,191 image tiles extracted from 39 WSIs. A subsequent application of a classical machine learning-based slide classifier sorted the WSIs into ‘low risk’ (benign, inflammation) and ‘high risk’ (dysplasia, malignancy) categories. We further trained the composite AI-model’s performance on a larger cohort of 105 resections WSIs and then validated our findings on a cohort of 150 biopsies WSIs against the classifications of two independently blinded pathologists. We evaluated the area under the receiver-operator characteristic curve (AUC) and other performance metrics. The AI model achieved an AUC of 0.917 in the validation cohort, with excellent sensitivity (97.4%) in detection of high risk features of dysplasia and malignancy. We demonstrate an unique composite AI-model incorporating both a glandular segmentation deep learning model and a classical machine learning classifier, with excellent sensitivity in picking up high risk colorectal features. As such, AI plays a role as a potential screening tool in assisting busy pathologists by outlining the dysplastic and malignant glands.

## Introduction

Artificial intelligence (AI) is rapidly revolutionizing the field of pathology^1,2^. Founded on the specialty practice of interpreting expressed histomorphological changes in cellular or tissue structure caused by disease processes, pathology has maintained its clinical utility till today^3^. The objective evaluation of histological slides by highly-trained pathologists remains the gold standard for cancer diagnosis^4^. With ever increasing workloads on pathologists, this time-consuming and manpower intensive work has recently seen the advent of computational pathology^5^ largely enabled by whole slide images (WSIs) which are digital counterparts of traditional glass slides and which have received selected FDA approval for primary clinical diagnosis^5,6^. Through application of medical image analysis, machine learning and deep convolutional neural networks (CNN), artificial intelligence have been used to inspect WSIs and produce computer-aided diagnosis (CAD) of cancers^1,5,7–9^. These CADs have demonstrated non-inferiority in the identification of malignancy compared to traditional means^8,10–12^.

While human pathologists can outperform such AI systems, they are subject to fatigue, time-constraints and observer bias in clinical settings. As such CNN has the additional benefit of unimpaired accuracy only subject to the operational capacity of its processing hardware. With ever-increasing workloads, the integration of artificial intelligence into the field of computational pathology is a growing necessity^8^. With an annual estimated 1.8 million cancer cases and 900,000 cancer deaths globally it places significant strain on healthcare systems^13,14^. The number needed to diagnose (NND) is further increased as some studies showed that as few as 7.4% of colonoscopy biopsies had any positive findings at all^15^. This means that an AI, which is capable of reliably screening the colonoscopy biopsies, could considerably reduce the workload of a practicing pathologist^16,17^. Additionally, it is worth noting that the assistive effect of AI on pathology is not limited to the evaluation of slides but extends to the acquisition of the target tissue of interest during sampling in a clinical setting such as narrow-band imaging^18–24^. If combined with in-vivo endoscopic assessments, AI can effectively revolutionize and streamline current diagnostic workflows^1,25^.

However, the development of CNN for colonic histopathology lags behind that for breast and prostate tissue^1,5,8^. We attempted to address these issues with our own CNN model by replacing outdated heatmaps and saliency maps with segmentation as the output^11,26,27^. Unlike other studies that typically rely purely on established datasets or open source segmentation architecture, we independently designed our own model which was trained and validated on separate training and validation sets^1,6,28^.

This successful application of a highly functional CNN to colonic biopsy WSI demonstrates the capability of AI in detecting epithelial tumours from a non-neoplastic background. This further shows that AI is very relevant in the specific field of colonic histopathology^5^. Expanding on these research developments, we describe our experience in this single-centre pilot study.

## Materials and methods

Our AI algorithm consisted of two distinct models. The first is a gland segmentation model that identifies potentially high risk regions on WSIs. The second involves a slide classification model that classifies WSIs as either ‘high risk’ or ‘low risk’. Using two separate models ensures robustness of the results and gives the operating pathologist more insight into the reasoning behind the slide classification. This allows for easier resolution of incongruent diagnoses and closer surveillance of the output data at this early stage of validation.

To produce these WSIs, our laboratory processed colonic specimens with haematoxylin and eosin (H&E) and scanned them with the Philips digital pathology whole-slide scanner, which satisfies current best practice recommendations^29^.

### Inclusion and exclusion criteria

We included samples from colon biopsies (May to June 2019) and resections (June 2019 to October 2019) obtained from Singapore General Hospital’s pathology archives and The Cancer Genome Atlas (TCGA). All slides from our institution were anonymized and de-identified H&E slides. We excluded all slides that did not contain mucosa, were poor in image quality (e.g., image being blurred, tissue being folded) or contained malignancies other than colonic adenocarcinoma. We also excluded slides suggestive of mucinous adenocarcinoma and signet ring carcinoma as these entities were poorly represented in our training data. This ensured proper robustness of our AI model in achieving our objective of detecting classic adenocarcinoma histology.

### Slide classifier

The gland segmentation model is a deep neural network that is built according to a Faster Region Based Convolutional Neural Network (Faster-RCNN) architecture for instance segmentation with a ResNet-101 feature extraction backbone. This is a standard architecture for segmentation tasks and many state-of-the-art instance segmentation models utilize this architecture or a variant thereof. The cost of this high accuracy complex model is a longer runtime. This is deemed as an acceptable trade-off for this device due to the relatively high importance of accuracy in the context of cancer detection. The slide classification model is a gradient-boosted decision tree classifier that uses the outputs of the gland segmentation model to classify slides as either high or low risk.

The training data for our gland segmentation model was extracted from WSIs. Ten WSIs were of colon resections obtained from TCGA, while 21 WSIs were of colon resections and 8 WSIs were of colon biopsies obtained from Singapore General Hospital.

**Figure 1:**
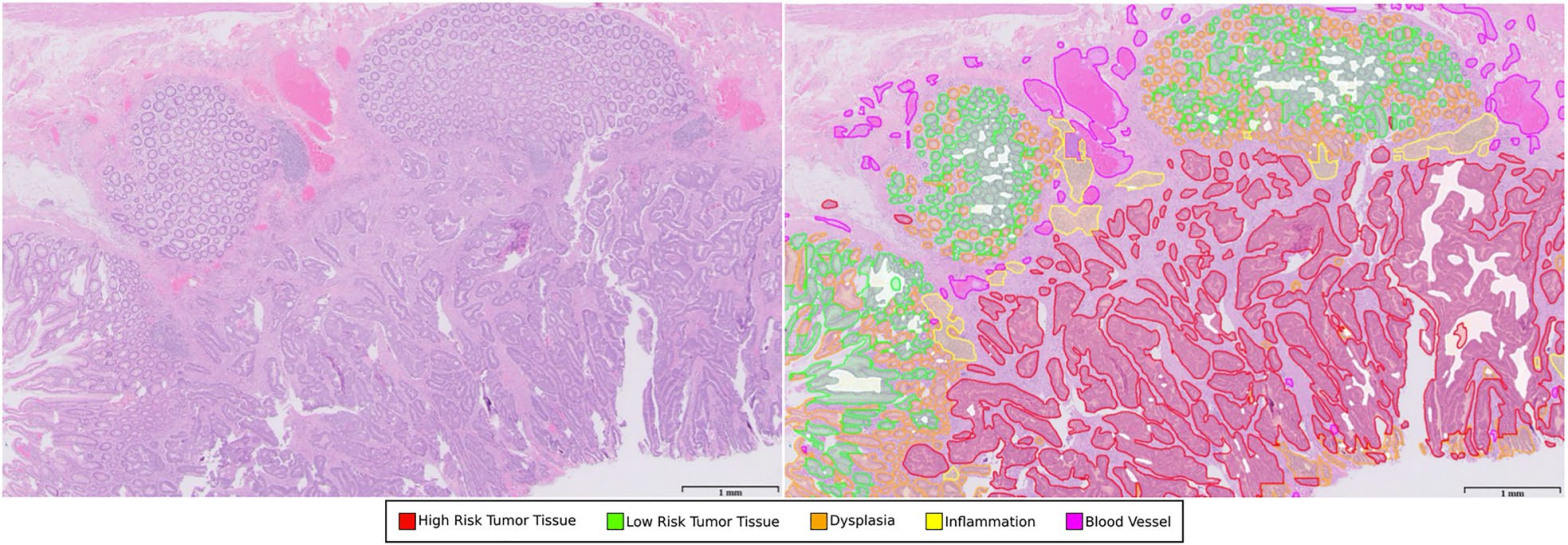
A sample analysis on a whole slide image (WSI). Left: original image. Right: the segmentation model highlighted regions of the WSI as (1) likely benign or normal (green), (2) likely dysplastic (orange), and (3) likely malignant (red). The AI model also segmented blood vessels (pink) and inflammation (yellow), and these segmentations were taken into account for slide labeling.

These slides underwent ground truth annotation by an expert pathologist studying the dataset. Slides were classified as either “high risk” or “low risk”. The WSIs were further annotated with labels from the following 7 categories: (1) benign glands, (2) glands that are either characteristic for adenocarcinoma or high-grade dysplasia, (3) low-grade dysplasia, (4) blood vessels, (5) necrosis, (6) mucin or (7) inflammation (Fig. 1).

### Training algorithm and protocols

To train our gland segmentation model, heuristic algorithms were used to identify tissue regions on WSIs. These were then divided into tiles of 775 × 522 pixels each. Tiles that did not contain any tissue (as determined by a tissue masking algorithm) were discarded. Likewise, ground truth annotations were separated into corresponding tiles so that each pixel was clearly labeled as either background or one of the categories described above. This resulted in a total of 73,546 image and ground truth tiles. Of these, 66,191 tiles (corresponding to 90% of all tiles) were used to train the neural network itself. We used the standard training paradigm for neural networks of backpropagation with stochastic gradient descent to adjust the parameters of the network. This is an iterative training procedure that gradually adjusts parameters to minimize a loss function. Five percent of all tiles (3678 tiles) were used for model selection. The neural network was trained with a constant learning rate of 0.016 over 15 epochs. We also performed data augmentation to artificially increase the training set and make the resulting model robust against predictable variations in data, such as imaging artifacts or variations in staining intensity. We have detailed the augmentations in Table 1. At regular intervals throughout the training, the model was evaluated on this development set. At the end of the training, the model parameters which resulted in the best performance on this development set were selected as the final model parameters. These yielded the final 3677 tiles (corresponding to 5% of all tiles) which were used to prevent overfitting.

**Table 1:** Data augmentations applied during training.

Augmentation method	Details
Rotation	Images were randomly rotated by 0°, 90°, 180°, or 270° with equal probability
Mirroring	Images were randomly mirrored along either the horizontal axis, the vertical axis, or neither axis with equal probability
Contrast	Increases or decreases the contrast of an image by shifting pixel values either away from or towards the mean intensity of the image, respectively, by a uniformly sampled random multiplicative factor between 0.7 and 1.3
Brightness	Increases or decreases the brightness of an image by increasing or decreasing the pixel values, respectively, by a uniformly sampled random multiplicative factor between 0.7 and 1.3
H&E color augmentation	The image is transformed from the RGB color space into the H&E color space so the first image channel now corresponds to the intensity of the hematoxylin and the second to the eosin stains. These two channels are now modulated by adding or subtracting uniformly sampled random numbers between − 0.05 and 0.05 to each channel. The image is then converted back to the RGB color space
	This augmentation is meant to simulate the effects of variability in the staining intensity
Gaussian noise	Every pixel is additively modulated by a normally distributed number (sigma = 0.1). This is meant to simulate various digital image artifacts that can occur during the scanning process
Gaussian blur	A Gaussian blur effect is applied to the entire image (radius = 0.1) to simulate microscope images that may be slightly out of focus

The slide classification model was trained with data comprising 105 WSIs of colon resection slides from Singapore General Hospital. As with the segmentation model, resection slides were intentionally chosen to train our model to ensure that the final device was able to generalize on new datasets and no bias was introduced into the feature selection at this stage. As tissue on colon resection slides are typically much larger and have different composition compared to colon biopsy slides, a model that performs equally well on both types of slides can be assumed to generalize well over new data.

**Figure 2:**
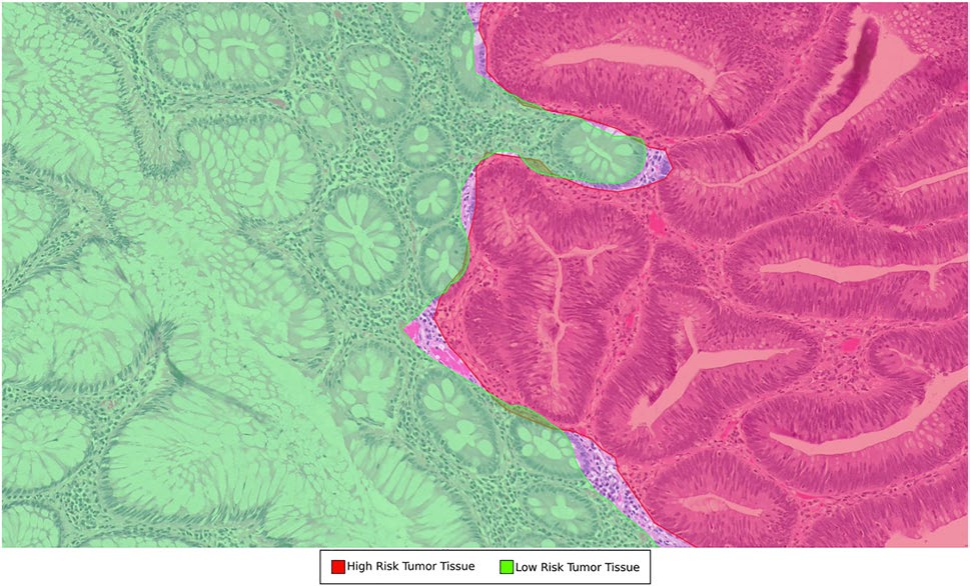
Labelling of “high-risk” (red) versus “low-risk” (green) regions based on the ground truth. A tile is labeled high-risk if there is overlap with any amount of high-risk ground truth annotations. Otherwise, the tile is labeled as low-risk. In this example, the highlighted tile would be labeled high-risk. The same rule was also applied to AI annotations. If a tile contains any amount of high-risk annotation given by AI, the tile is labeled as high-risk by AI, and low-risk otherwise.

The ground truth annotations used in the training dataset for the slide classification model was created by two pathologists analyzing the WSIs. Slides were classified as either “high risk” (contains sign of adenocarcinoma or dysplasia) or “low risk” (contains normal histology/inflammation/reactive changes without signs of adenocarcinoma or dysplasia). Disagreements between pathologists were solved through discussion. Upon agreement, this final diagnosis was utilized as the ground truth (Fig. 2).

### Validation protocols

The fully trained segmentation model was applied to all 105 WSIs. The segmentation results were aggregated to obtain numeric features for each slide. Features were selected from an ensemble of potential features based on their relative impact on the slide classification. These were: (1) the total area classified as either adenocarcinoma or dysplasia by the segmentation model with a prediction certainty of greater than 70% (this area is expressed as a percentage of the total tissue), (2) the average prediction certainty for adenocarcinoma or dysplasia objects weighted by their areas, (3) an additional Boolean flag that was set to 1 if the slide had adenocarcinoma or dysplasia predictions with a prediction certainty of greater than 85% that cover area of at least 0.1 mm^2^, and (4) the bottom 1-percentile of adenocarcinoma or dysplasia predictions weighted by their areas. Once these numeric features were obtained, fivefold cross validation was applied and an ensemble of models was trained, tuned, and compared. The model and hyperparameter combination with the optimal cross validation performance, a gradient boosting classifier, was selected. Inputs to these models were the features described in the previous step and the output was a binary classification into high risk or low risk. The highest performing model with greatest accuracy was selected to be trained and finalized on the entire dataset consisting of 105 WSIs.

### Declarations

We confirm that all methods were carried out in accordance with relevant guidelines and regulations. All experimental protocols were approved by the SingHealth Centralized Institutional Review Board. Informed consent was obtained from all subjects and/or their legal guardian(s) prior to having their data used in this study.

## Results

The composite AI model, consisting of the gland segmentation and slide classification, was fully trained on the 105 resection WSIs and validated on the separate dataset of 150 biopsy WSIs. The biopsy WSIs were classified into high risk and low risk, similar to the resection slides used for training the slide classifier. After obtaining the output labels produced by the AI algorithm from the validation set, we compared them to the ground truth labels produced by our expert pathologists. We calculated the number of true positives, true negatives, false positives, and false negatives. Using these statistics, we then calculated several performance metrics, such as the sensitivity, specificity, and area under the receiver-operator characteristic curve (AUC).

**Figure 3:**
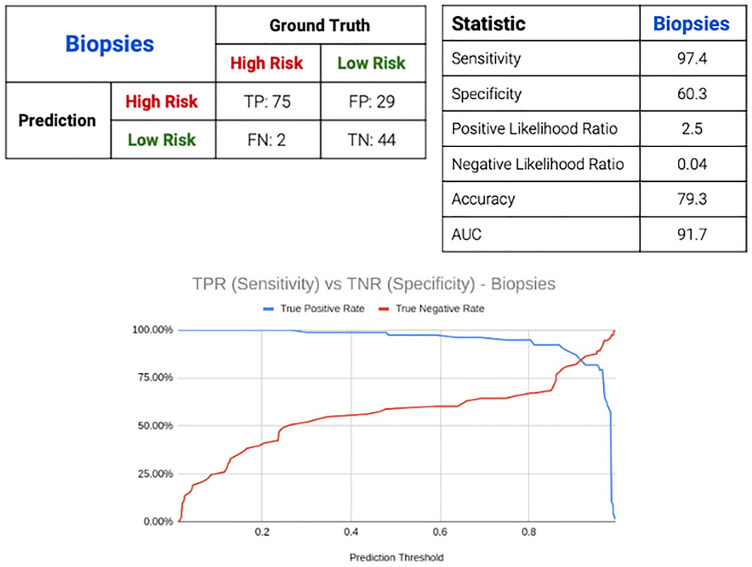
Performance data from applying the AI model on the validation set of 150 WSIs.

**Figure 4:**
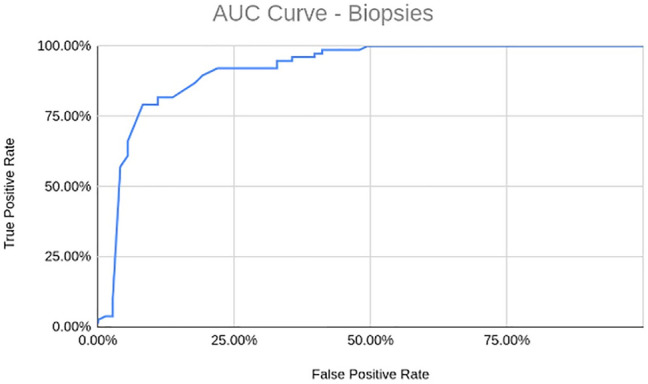
AUC curve from applying the AI model on the validation set of 150 WSIs. The system achieves an AUC of 91.7%.

Our AI model classified 119 of the 150 biopsies correctly. There were 31 errors consisting of 2 false negatives and 29 false positives. With these, the AI model achieved high sensitivity of 97.4% and lower specificity of of 60.3 with an AUC of 91.7 (Fig. 3). These performance metrics and trade-off between sensitivity and specificity is demonstrated in Fig. 4. By changing the prediction threshold to values above 0.7, we could obtain both sensitivity and specificity values above 80%. This prediction threshold refers to the probability cut-off in which the AI classifies a slide as either high risk or low risk. We deliberately set this threshold of 0.7 to suit the operation needs of our institution. Such a threshold allows the model to achieve greater sensitivity in detection of malignancy and functioning as a triage system. The prediction threshold can be adjusted easily to suit other operational requirements as the user deems appropriate.

## Discussion

**Figure 5:**
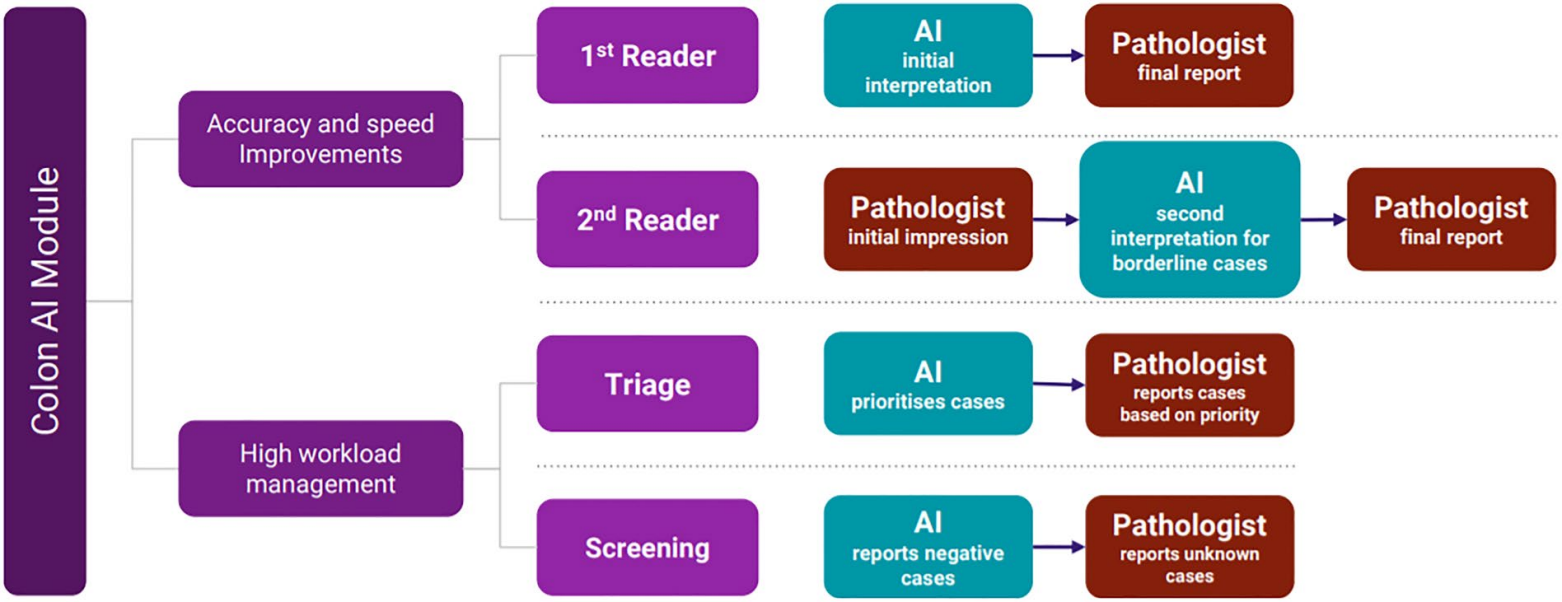
Proposed workflow for the use of AI in pathology.

Upon evaluation of the output data from the validation set, its high AUC of 91.7 demonstrates a good concordance between the AI model and the expert pathologist. With a sensitivity of 97.4, it validates our AI model to function as a screening tool that minimizes false negatives extensively. This favour of sensitivity over specificity shows its usability in assistive workflow and has added practicality into the application of a clinical workflow. Since our AI model is designed solely as an assistive tool, the final diagnosis during reporting remains with the pathologist. This high sensitivity ensures with greater certainty that all lesions suspicious for malignancy are highlighted for further pathological review. This triage system is part of a diagnostic workflow we recommend the AI model be implemented in. We also propose four other possible ways in which AI model could be incorporated in pathology workflow; it could act as first reader, second reader, triage, and pre-screening (Fig. 5)^10,30,31^.

Although multiple studies have reported the role of AI in colorectal polyp detection and characterization, our study is unique in the usage of segmentation as the output. The prevailing trends in the literature are based mainly on heat maps or saliency maps^9,26,32^. We believe that segmentation offers significant advantages over heatmaps. In segmentation, WSIs are split apart into small patches which are analysed by an AI algorithm. These segmentation-based annotations are individually more detailed, have more intuitive presentation of results to pathologists and enable pathologists to derive more insightful quantitative information from the WSIs segment^11,27^. There is also more explicability behind prediction made by the system. Segmentation also allows for calculation of tumor area and higher potential for biomarker discovery while allowing pre-annotation of whole-slide images using AI^33^. Finally, it is also more versatile and is able to segregate more elements on the slide, which means we can view multiple parameters on a single image^1^. For example, in addition to being able to segment the colonic tissue into three main categories: likely benign or normal (green), likely dysplastic (orange), and likely malignant (red), our AI system also segments additional structures such as blood vessels (pink) and inflammation (yellow) (Fig. 1). Additional parameters can be easily programmed into our algorithm, allowing for more detailed information.

By having separate training and validation sets, we have avoided overfitting and reduced bias in our study. We have also compared our F1 scores over existing segmentation algorithms with similar convolutional neural network architecture (Table 2). This composite metric is based on precision and recall sameness which directly correlates to the product of sensitivity and specificity in the AI model^25,27^. The vast majority of current AI studies utilize fully-supervised learning based on established datasets which are available as open-source data^1,25,27^. There are few similar studies that have presented an AI algorithms based on an architecture consisting of two independently trained models, though an attempt has been made to separately segment glands from the background to identify gland edges^1,7^. There has also been the development of a neural network architecture that is similar to but less efficient than the RCNN architecture which is more commonly used in instance segmentation tasks^34^. This architecture consisted of three arms—one to separate glands from the background, one to identify gland edges, and a third one to perform instance bounding box detection.

**Table 2:** Comparison of studies with similar convolutional neural network architecture.

Study	Objectives	Dataset	F1 score
Xu et al. (2017)	Segmentation of colon glands	GLAS challenge (165 images)	0.893**–**0.843
Xu et al. (2016)	Nuclei segmentation	537 images from Case Western Reserve University	0.858**–**0.771
Korbar et al. (2017)	Deep Neural Network Visualization to Interpret WSI Analysis Outcomes for Colorectal Polyps	176 WSIs from Dartmouth-Hitchcock Medical Center	0.925**–**0.841
MIMO—Net^35^	Various studies	Various studies	0.913**–**0.724
DeepLab v3+^36^	Various studies	Various studies	0.862**–**0.764
SegNet^37^	Various studies	Various studies	0.858**–**0.783
FCN—8^37^	Various studies	Various studies	0.783**–**0.692
Qritive Colon AI (current study)	Glandular segmentation deep learning model to detect high risk colorectal polyps	WSIs produced from 294 colorectal specimens from Singapore General Hospital	0.974–0.856

*GLAS* Gland Segmentation in Colon Histology Images Challenge Contest, *WSI* whole slide images.

Our study was not without limitations. Our small sample size is susceptible to heterogenicity of data and that could hence diminish it’s applicability to clinical practice in screening a general population. Further studies on a larger dataset are underway. Additionally, while the performance data from the validation had a high AUC of 91.7, it still contained 2 false negatives but 29 false positives. This was largely attributable to the manipulation of the prediction threshold values. In a clinical institution with AI model being applied to patient care in the context of cancer diagnosis, the system was designed to favour sensitivity over specificity to ensure usability in assistive workflow. This allows the AI model to categorize all high grade dysplasia and adenocarcinoma as ‘high risk’. This carry-over effect, when applied onto the validation set, resulted in higher false positives as the prediction certainty of the various features were forced into a binary classification, leading to images with relatively low prediction certainty of 70% being highlighted as ‘high risk’.

The issue of false positives can be addressed by further training and validation of the composite AI model. It is estimated that at least 10,000 WSIs are required to train a weakly supervised AI model for histopathology even before accounting for the lack of the heterogeneity encountered in WSIs with regard to staining, anomalies, tissue textural variation and polymorphism^12,33,38^. Our dataset will need further expansion to a much larger sample size and conduct a large-scale multi-site clinical validation to improve the quality of our AI model to reduce misclassification rate and achieve better results. Ideally, we intend to eventually remove the machine learning classifier and transition entirely into a gland segmentation model. The machine classifier was added to avoid overfitting as we expand on our current dataset. Currently, two separate models provide more insight into the reasoning behind the slide classification. This allows for easier deconflicting of incongruent diagnoses and closer surveillance of the output data as we further refine our AI algorithm.

Additionally, current segmentation output models utilise the most frequent tile classification output as the resulting predicted slide label and aggregated to produce a slide-level output but this provides limited contextual knowledge with a narrow field of view, while grossly increasing computational complexity^39–43^. Such patch-based models are not consistent with the manner pathologists analyse slides under microscopes and fail to take into consideration the characteristics of the surrounding structures and the overall morphology of the WSI^1^. While approaches to alleviate this issue such as designing multi-magnification networks exist, they have largely been limited^44^. Some authors have proposed a variant of a fully conventional network (FCN) which consists of dense scanning, anchor layers, or combining FCNs with CNNs^41,42^.

Furthermore, more work can be done in regard to pre-processing of slides for increased standardisation, including, but not limited to, stain normalization, augmentation and stain transfer^1^. While our laboratory’s process of H&E staining and scanning with the Philips digital pathology whole-slide scanner satisfies current best practice recommendations, it is worth noting that earlier works in digital pathology had previously assumed staining attenuated optical density and applied uniformity assumptions^29^. However, this has fallen out of favour with chemical staining and tissue morphology now being considered in the generation of stain transfer matrices^45–48^.

## Conclusion

In summary, we demonstrated that our unique composite AI model incorporating a glandular segmentation deep learning model and a machine learning classifier has promising ability in picking up high risk colorectal features. The high sensitivity highlighted its role as a potential screening tool to create initial interpretations and assist pathologists to streamline the workflow, thereby effectively reducing the diagnostic burden on pathologists. Ongoing calibration and training of our composite AI model will improve its accuracy in risk classification of colorectal specimens.
